# A reassessment of the energetic significance of blood lactate accumulation during exercise

**DOI:** 10.1007/s00421-026-06134-8

**Published:** 2026-02-12

**Authors:** Guido Ferretti, Pietro Enrico di Prampero

**Affiliations:** 1 Department of Molecular and Translational Medicine, University of Brescia, Viale Europa 11, Brescia, 25123 Italy; 2 Istituto Lombardo Accademia di Scienze e Lettere, Milano, Italy; 3 Emeritus Professor of Physiology, University of Udine, Udine, Italy

**Keywords:** Muscle, Glycolysis, Pyruvate, Anaerobic power, Energy equivalent of lactate

## Abstract

In this perspective paper, we reassess the theoretical bases and reanalyse the experimental evidence supporting the concept that blood lactate accumulation during exercise at powers above the maximal lactate steady state corresponds to and reflects the metabolic power derived from anaerobic lactic metabolism. We discuss the biochemical background of anaerobic lactic metabolism and the direct proportionality between the anaerobic lactic metabolic power and the corresponding rate of lactate accumulation. We compute the energy equivalent of lactate accumulation, defined as the slope of such relationship. We then discuss lactate distribution and its impact on lactate washout during recovery at the end of exercise and the concept of lactate threshold within the present energetic perspective. We eventually explain why we can have higher yet stable lactate concentrations at exercise than those prevailing at rest and we discuss the energetic significance of lactate accumulation during the exercise transients (early lactate). Our aim is to convince readers that a simple measure of blood lactate is sufficient to obtain valuable information on the power generated by anaerobic lactic metabolism.

## Introduction

Recently, Taboni et al. (2025a) demonstrated, in this Journal, that lactate is not a direct cause of the slow component of oxygen uptake kinetics. That article generated a letter by Poole and Gaesser (2025), in which the authors claimed that they also attained similar conclusions through other, more indirect and correlative ways. This is fair and the authors’ response (Taboni et al. 2025b) easily acknowledged it. Yet, a sentence in Poole and Gaesser letter attracted our attention, the last one, namely: *Endogenous lactate accumulation should*,* perhaps*,* be considered as a stress response to a certain metabolic strain evoked by intramyocyte physicochemical perturbations rather than an “anaerobic” process or substantive energy source during heavy or severe-intensity exercise.* This is a surprising sentence, since the rate at which lactate accumulation takes place in muscle and in blood is the epiphenomenon highlighting the occurrence of anaerobic lactic metabolism in working muscles during exercise. As such, that sentence reveals some misunderstanding of the energetic significance of lactate accumulation, suggesting that our previous discussions and analyses of anaerobic metabolism were not clear enough about the energetic role of lactate, and thus not sufficiently convincing (di Prampero 1981; di Prampero and Ferretti 1999; Ferretti 2015). Hence, we think that the time has come to reassess and clarify the concepts of anaerobic lactic metabolism and of its energetic equivalent.

Before entering into details, we would like to make it clear that this perspective paper concerns only the energetic significance of the rate of lactate increase with time during exercise as an index of ongoing anaerobic metabolism. Therefore, we do not exclude other possible biological and physiological roles of muscle and blood lactate accumulation, in particular as signalling molecule, for which we address the readers to other narrative reviews on those topics (Halestrap 2013; Brooks 2018; Rabinowitz and Enerbäck 2020; Benitez Muñoz and Cupeiro 2025).

## Biochemical considerations

Glycolysis is the biochemical process by which cells transform glucose into pyruvate through a series of chain reactions. Embden et al. (1933) eventually solved the puzzle of the overall glycolytic pathway, which therefore is well known since almost a century. Pyruvate has two possible fates: either (i) it forms acetyl-coenzime A, which feeds the Krebs cycle, or (ii) it is reduced to lactate by the action of lactate dehydrogenase (LDH). In mammals, the former is the case when the respiratory system provides enough oxygen to keep the rate of aerobic metabolism (Krebs cycle and oxidative phosphorylation) matched to the rate of glycolysis. The latter occurs when the rate of glycolysis exceeds that of the aerobic metabolic pathway, so that pyruvate formation exceeds pyruvate removal through the Krebs cycle. Therefore, pyruvate tends to accumulate in the contracting muscles and the equilibrium of the LDH reaction is displaced toward the formation of lactate, which also accumulates in muscles: this is when anaerobic lactic metabolism starts.

However, lactate, formed e.g. in a type II muscle fibre, can also be taken up by an adjacent type I fibre, re-oxidized to pyruvate and eventually washed out by the Krebs cycle in the latter fibre. In other words, this process is tantamount to Brooks’ concept of lactate shuttle across two muscle fibres (Brooks 2009). In this case, we still have aerobic metabolism going on, although across two adjacent fibres. Obviously, the displacement of lactate from the former to the latter fibre requires an increase in lactate concentration ([*La*]) in the active muscle, yet, once the equilibrium has been attained, the process is fully aerobic and [*La*], even if higher than at rest, remains invariant in time. Indeed, we have anaerobic lactic metabolism only if [*La*] keeps increasing with time.

The ratios at which chemical transformations occur in glycolysis are simple and constant, according to the principle of Avogadro. In the case of anaerobic metabolism, we have the net formation of two units of pyruvate and of lactate and three units of ATP from the progressive oxidation of one unit of glucose through glycolysis, whence a theoretical phosphate/lactate ratio (P/La ratio) of 1.5, which is a stoichiometric constant. In physiological conditions, from data obtained on isolated muscles (di Prampero et al. 1978; Cerretelli and di Prampero 1988), a P/La ratio ranging between 1.25 and 1.05 could be calculated (di Prampero and Ferretti 1999). These figures, not far from theoretical predictions, imply either a slight underestimate of the work done per unit of lactate formed or a light overestimate of the work done per unit of ATP split or both.

The work done by a unit of ATP split is also a constant, depending on ATP concentration. Its value, determined on isolated frog muscle and on the isolated-perfused dog muscle preparation, and estimated during explosive efforts in humans, ranges between 16 and 19 kJ mol^− 1^ (Cerretelli et al. 1969; Ferretti et al. 1987; Cerretelli and di Prampero 1988). Therefore, the mechanical efficiency of ATP splitting into work is a constant as well, and so is the enthalpy change (work plus heat) that is generated by the splitting of a unit of ATP.

## Of anaerobic lactic metabolism

It follows from what precedes that (i) the accumulation of a lactate unit in contracting muscles generates a constant work and a constant amount of metabolic energy to be stocked in muscle as ATP, and (ii) the rate at which [*La*] increases ($\:\frac{dLa}{dt}$) is proportional to the metabolic power provided by anaerobic lactic metabolism ($\:\frac{{dE}_{La}}{dt}$). Thus, we can write:

1 $$\:\frac{{dE}_{La}}{dt}=\beta\:\frac{dLa}{dt}$$

where $\:\beta\:$ is a proportionality constant indicating the amount of energy derived from the accumulation of a unit of lactate, *id est*:

2 $$\:\beta\:=\frac{{dE}_{La}}{dLa}$$

If $\:dLa$ is expressed in mmoles and $\:{dE}_{La}$ in Joules, $\:\beta\:$ is in Joules mmoles^− 1^. Of course, we can convert this unit into any other equivalent unit. In exercise physiology, it is customary to report lactate as a concentration in a fluid. Lactate is generally measured in venous blood, which becomes the reference fluid. $\:{dE}_{La}$ instead is often expressed in oxygen equivalents, assuming that, when only glucose is used as substrate, 1 ml of oxygen provides 21.16 Joules of metabolic energy. Constant $\:\beta\:$ is named the energy equivalent of lactate accumulation. It was determined under various circumstances and a value ranging between 2.7 and 3.0 ml of oxygen per kg of body mass per mmole of lactate was always obtained (Margaria et al. 1963; Pendergast et al. 1977; Capelli and di Prampero 1995; Lador et al. 2013; Ferretti 2023; Borrelli et al. 2024)^1^. Taboni et al. (2025a) used a midway value in this range to demonstrate deductively that lactate is not a cause of the slow component of the oxygen uptake kinetics upon exercise onset. Of course, defining a range rather than an exact value for $\:\beta\:$ results from an inevitable variability of measurement, depending on the experimental conditions and on the intrinsic errors of measurements, as always is the case when dealing with a ratio of two linear slopes. Moreover, in practice, capillary, venous, and arterial [*La*] can differ among them, and the active muscle mass is not an invariant fraction of the body mass within the human population. Yet the reported range is amazingly narrow, suggesting remarkable repeatability of $\:\beta\:$ determination in a wide variety of conditions, despite the potential measurement errors.

When during exercise a [*La*] higher than resting stays invariant in time (Ribeiro et al. 1986; Nixon et al., 2021; Almeida Azevedo et al. 2022), the rate of lactate increase is nil and there is no anaerobic lactic metabolism going on. On this basis, Beneke (2003) introduced the concept of maximal lactate steady state (MLSS), defined as the highest [*La*] that can be maintained stable during exercise for at least 10–15 min. During supramaximal exercise (Margaria et al. 1963, 1964; Pendergast et al. 1977; Capelli and di Prampero 1995; Grassi et al. 1995), or at least during exercise at constant powers above the MLSS (Beneke and Von Duvillard 1996; Beneke et al. 2003; Nixon et al., 2021), [*La*] increases linearly with time. This means that, at constant mechanical power, the rate at which [*La*] increases in time is invariant (constant $\:\frac{dLa}{dt}$) and so is the corresponding metabolic power (constant $\:\frac{{dE}_{La}}{dt}$). The higher is $\:\frac{dLa}{dt}$ – slope of the linear relationship between [*La*] and time above the MLSS – the higher is $\:\frac{{dE}_{La}}{dt}$ in direct proportion.

Equations 1 and 2 and the underlying reasoning are coherent with the first principle of thermodynamics. The chemical energy liberated during ATP splitting is equal to the enthalpy (work plus heat) generated in muscle contraction by the ATP-ase activity of myosin. The amount of chemical energy stored as ATP in anaerobic glycolysis leading to lactate accumulation is proportional to the net number of ATP units formed during glycolysis. This amount has a precise and fix stoichiometric relationship to the units of lactate that are formed. An increase in [*La*] is therefore proportional to the amount of energy stocked in the process. Moreover, the rate at which lactate is accumulated, no matter whether in muscle or in blood, is directly proportional to the metabolic power generated in the process.

The experimental evidence quoted above supports these concepts. At any given power during intense exercise, [*La*] increases linearly with time, as does $\:{E}_{La}$; moreover, $\:\frac{{dE}_{La}}{dt}$ increases linearly with the mechanical power, as does $\:\frac{dLa}{dt}$, so that the relationship between $\:\frac{{dE}_{La}}{dt}$ and $\:\frac{dLa}{dt}$ is linear, its slope being the energy equivalent of lactate accumulation $\:\beta\:$, which is a constant. $\:\beta\:$ is therefore independent of time, and thus of the time of lactate distribution in the body from the contracting muscles. As soon as, at any given power, blood [*La*] starts increasing linearly with time, the equilibrium is attained with lactate accumulation in the working muscles and with the corresponding $\:\frac{{dE}_{La}}{dt}$. The occurrence of a time delay for lactate accumulation between muscle and blood, which is usually sampled from an arm, an earlobe or a fingertip, thus far from the working muscles, becomes irrelevant, because time is not part of the computation of $\:\beta\:$.

To our understanding, there is only a condition in which constant $\:\beta\:$ may become higher than reported here above. The uppermost limit of 19 kJ mol^− 1^ for the work done by a unit of ATP split is outreached when ATP concentration in muscle fibres becomes lower than 4 mM. If such an increase in the work done by a unit of ATP split is not balanced by a reduction of heat delivery (increase of mechanical efficiency), also the enthalpy released by ATP splitting would increase. In this case,$\:\:\beta\:$ may turn out higher than 3.0 ml kg^− 1^ kg mmol^− 1^. However, this is quite an unlike situation indeed, because the concentration of ATP in muscle fibres is kept invariant over a wide range of phosphocreatine concentration values, because the equilibrium constant of Lohmann reaction ranges between 20 and 100, depending on pH and magnesium concentration (Carlson and Siger 1960; Canfield and Maréchal 1973).

## Of lactate distribution

Although the issue of lactate distribution does not concern the computation of $\:\beta\:$, yet it was used in the past to criticize the energetic significance of blood lactate accumulation (Karlsson 1971; Åstrand and Rodahl 1977). Discrepancies between muscle [*La*], determined on muscle biopsy samples, and peak blood [*La*] at the end of maximal exercise of short duration were at the basis of these criticisms. These discrepancies appear obvious, considering the short duration of the exercise at stake and the time delay between muscle lactate increase and a distant blood lactate increase. When lactate accumulates and energy generation by anaerobic metabolism occurs, blood [*La*] is always lower than working muscles [*La*], and in a predictable way depending on the time lactate takes to cover the distance between working muscles and site of blood sampling. Yet, as already pointed out, once lactate starts increasing linearly with time also in blood, an equilibrium between $\:\frac{dLa}{dt}$ in muscle and in blood is attained and the slope of the two relationships is necessarily the same. This being so, it is legitimate to compute $\:\beta\:$ from blood lactate measurements.

Differences in the time course of blood and muscle [*La*] become important in the analysis of the kinetics of lactate removal during recovery after the end of exercise. In the early phase of recovery, lactate in blood is still increasing while lactate in muscles is already declining. Only after attainment of a peak [*La*] in recovery, usually within 3 min, lactate starts decreasing also in blood. Then, the decrease follows a monoexponential trend, as originally demonstrated already by Margaria et al. (1933). This indicates that the ensemble of body fluids, not only blood, acts as a single capacitance of large size (half-time around 15 min in resting recovery). A convincing evidence supporting Margaria’s finding in humans was collected in the past (Margaria et al. 1963; Gisolfi et al. 1966; Hermansen and Stensvold 1972; Belcastro and Bonen 1975; Dodd et al. 1984).

## Of lactate threshold MLSS and critical power

The concept of lactate threshold (LT), in its various definitions, identifies a specific blood [*La*] above which anaerobic lactic metabolism is claimed to appear. This [*La*] is identified on plots in which [*La*] is expressed as a function of mechanical power. Above the LT, any increase in [*La*] is associated with hyperventilation. Despite claims, from an energetic perspective, the LT has nothing to do with the onset of anaerobic lactic metabolism. On the LT plot, the ordinate carries [*La*], no matter whether lactate is varying in time or not. However, as highlighted above, anaerobic lactic metabolism occurs only if [*La*] is continuously increasing with time, that is only if $\:\frac{{dE}_{La}}{dt}$ and $\:\frac{dLa}{dt}$ have positive values. This may not be the case even at powers above the LT, conventionally set at 2 or 4 mM. For instance, Ribeiro et al. (1986) reported steady [*La*] above 5 mM: there was no anaerobic lactic metabolism in that case. A steady [*La*] indicates an entirely aerobic metabolism. Thus, no threshold indicating a shift to or an appearance of anaerobic lactic metabolism at the LT. From an energetic viewpoint, it is a meaningless concept. Nevertheless, the LT still represents a useful marker as a pragmatic threshold for exercise prescription and monitoring. In sport, the LT is commonly used in predicting performances in long distance running.

More significant appears the concept of MLSS, introduced by Beneke and Duvillard (1996) and formalised by Beneke (2003). From a qualitative standpoint, the MLSS is similar to the LT, yet with an important difference: whereas determination of the LT relies on a single value of [*La*], determination of MLSS requires multiple lactate measurements at constant power, demonstrating invariant [*La*] in time. The power at the MLSS is the highest power at which, once a steady state for oxygen consumption ($\:\dot{V}{O}_{2}$) has been attained, [*La*] does not vary in time, so that glycolytic lactate production is equal to oxidative lactate removal and the metabolism is fully aerobic. The definition of MLSS is compatible with the energetic principles outlined above, since, above the power corresponding to the MLSS, lactate appearance exceeds lactate removal and anaerobic metabolism flanks and sustains aerobic metabolism.

In a way, we prefer to suggest that the MLSS is somehow linked to the critical power (CP, Pringle and Jones 2002; Jones et al., 2010; Ferretti 2015; Leo et al. 2022), although not synonymous of it (Jones et al. 2019). The CP was originally defined in qualitative terms, as the highest power that an exercising individual can maintain for a very long time without fatigue (Monod and Scherrer 1965). This definition was retained afterward (Poole et al. 1988; Jones et al., 2010; Ferretti 2015) and is still accepted nowadays. The CP was initially associated to the LT (Moritani et al. 1981). This association assumes that the LT defines the boundary between aerobic and anaerobic metabolism, which is not correct from an energetic viewpoint. In fact, the demonstration that CP is higher than LT came soon (Poole et al. 1988, 1990), shortly after the demonstration that higher constant lactate levels than the LT may occur in prolonged exercise (Ribeiro et al. 1986). The subsequent association of CP with the MLSS, once this concept had been introduced (Beneke and Duvillard 1996), is a logical consequence of this state of things (Pringle and Jones 2002). A further step was the association of the so-called energy store component – the mathematical constant characterising the curvature of the hyperbolic CP model (Morton 1996; Jones et al., 2010; Ferretti 2015) – with anaerobic lactic capacity (Pringle and Jones 2002). In fact, the latter association is debatable, as long as the $\:\dot{V}{O}_{2}$ at the CP is lower than the maximal $\:\dot{V}{O}_{2}$. Thus, the energetics of the energy store component, which is the energy balance sustaining exercise above the CP up to maximal $\:\dot{V}{O}_{2}$, includes a mixture of aerobic and anaerobic lactic energy delivery (Ferretti 2015).

The mechanisms maintaining a steady [*La*] higher than resting have to do with the lactate shuttle hypothesis of George Brooks (Brooks 1986, 2000, 2009) and were outlined in energetic terms by di Prampero and Ferretti (2023). These mechanisms do not affect the value attributed to constant $\:\beta\:$. The MLSS, as defined above, represents the highest power at which $\:\frac{{dE}_{La}}{dt}$ is nil, but does not participate in the definition of $\:\beta\:$, which is the ratio of the slopes of two positive linear relationships, as pointed out above. The power at the MLSS is modified by the type of exercise, and thus by the active muscle mass, by muscle fibre composition and by the training status. In trained endurance athletes, who are characterised by an elevated fraction of type I muscle fibres in lower limb muscles, it is closer to the maximal power than in non-athletic individuals (Billat et al. 2003).

## Of early lactate

Where does then the increase in lactate, leading to a new steady lactate level during exercise at powers lower than the MLSS, come from? It comes from early lactate (Cerretelli et al., 1979), defined as the amount of lactate accumulated during the exercise transient and computed as the lactate accumulated in the first 5 min of exercise (Billat et al. 2003), before the attainment of the metabolic steady state (in fact, at powers close to the MLSS, a constant [*La*] concentration is attained within 10 min).

The kinetics of metabolic activation in the working muscles is coupled with that of respiratory oxygen flow ($\:{\dot{V}}_{R}{O}_{2}$). Together, they dictate the kinetics of activation of oxidative phosphorylation, and thus of muscle oxygen consumption ($\:{\dot{V}}_{m}{O}_{2}$). The kinetics of glycolytic activation is a mirror image of the kinetics of phosphocreatine splitting, which has a key role in regulating glycolysis and which provides the energy sustaining the anaerobic alactic component of the oxygen deficit (di Prampero 1981; Ferretti 2015). The kinetics of phosphocreatine splitting was modelled by di Prampero and Margaria (1968). Their predictions in humans were supported by experiments carried out using NMR spectroscopy, which provided a time constant for the monoexponential phosphocreatine decrease upon exercise onset of 23–25 s (Binzoni et al. 1992; Rossiter et al. 1999; di Prampero et al. 2003), independent of the exercise intensity (Binzoni et al. 1997). The kinetics of $\:{\dot{V}}_{R}{O}_{2}$ was determined in a huge amount of studies. After introduction of dual exponential models of $\:{\dot{V}}_{R}{O}_{2}$ kinetics and the progressive successive refinement of data analysis techniques, the time constant of the $\:{\dot{V}}_{R}{O}_{2}$ kinetics (of the primary component of $\:{\dot{V}}_{R}{O}_{2}$ kinetics, if the dual exponential model is used) at light exercise resulted faster than that of phosphocreatine splitting (Cautero et al. 2002; Lador et al. 2006). When this is so, the respiratory system provides enough oxygen to the working muscles to sustain pyruvate removal by aerobic metabolism through the Krebs cycle and oxidative phosphorylation: the time constant of $\:{\dot{V}}_{m}{O}_{2}$ results equal to that of phosphocreatine splitting. Thus, phosphocreatine splitting sustains the oxygen deficit entirely, and no lactate is accumulated during the exercise transient.

However, the time constant of the primary component of $\:{\dot{V}}_{R}{O}_{2}$ kinetics increases with the exercise intensity, as if the capacity of the respiratory system was expanding. When the rate of $\:{\dot{V}}_{R}{O}_{2}$ increase in the exercise transient becomes slower than that of phosphocreatine splitting, the matching of respiratory system and muscle metabolic activity is broken (Ferretti et al. 2023). In this case, the $\:{\dot{V}}_{R}{O}_{2}$ and thus the $\:{\dot{V}}_{m}{O}_{2}$ kinetics become slower than that of phosphocreatine splitting. The respiratory system provides less oxygen than needed to sustain aerobic metabolism in the exercise transient and the glycolytic pathway is accelerated at a faster rate than oxidative phosphorylation. Therefore, pyruvate tends to accumulate in the working muscles, the exceeding pyruvate is reduced to lactate by LDH, and lactate accumulates during the exercise transient (early lactate). If the power at which this occurs is lower than that at the MLSS, a new equilibrium at a new apparent [*La*] steady state is attained at a higher [*La*] than resting. The increase in [*La*] is proportional to the energy provided by early lactate.

Ferretti (2023) carried out an analysis of the energetics of the oxygen deficit component due to early lactate. To do this, he used data obtained in hypoxia, characterised by a longer time constant of $\:{\dot{V}}_{R}{O}_{2}$ kinetics than in normoxia at the same power (Springer et al. 1991; Hughson and Kowalchuk 1995; Engelen et al. 1996; Lador et al. 2013), namely those by Lador et al. (2013), the only who reported also early lactate values. These authors found no increase in blood [*La*] during light exercise in normoxia. Their estimated time constant of $\:{\dot{V}}_{m}{O}_{2}$ kinetics, after correction for changes in oxygen stores, corresponded well to that of muscle phosphocreatine kinetics (Binzoni et al. 1992).

This was not so in hypoxia, when the time constant of the $\:{\dot{V}}_{R}{O}_{2}$ kinetics resulted longer than that of phosphocreatine splitting, and the corresponding oxygen deficit larger than in normoxia. Since the mechanical power was the same in both cases, and the time constant of phosphocreatine splitting was therefore equal in both conditions, the higher oxygen deficit in hypoxia could be due only to an increase in early lactate. Thus, the energy provided by early lactate accumulation in hypoxia must be equal to the difference in oxygen deficit between hypoxia and normoxia. If this is so, the ratio between this difference and the net average lactate accumulation reported by Lador et al. (2013) must correspond to $\:\beta\:$. Indeed, when on these premises Ferretti (2023) calculated $\:\beta\:$ from the data of Lador et al. (2013), he obtained a value equal to 2.5 mlO_2_ mM^− 1^ kg^− 1^, just below the lower limit of the range defined above. Cerretelli et al. (1979) reported a linear increase of the half time of the $\:{\dot{V}}_{R}{O}_{2}$ kinetics with early lactate at constant power, allowing computation of the overall oxygen deficit. On the same premises of Ferretti (2023), a value for $\:\beta\:$ of 2.7 mlO_2_ mM^− 1^ kg^− 1^ was obtained. This analysis demonstrates that early lactate can indeed be the cause of the higher and steady [*La*] observed below the MLSS.

## Conclusions

In conclusion, we can state that:

1. Anaerobic lactic metabolism occurs only when [*La*] keeps increasing with time.
2. The energy equivalent of lactate accumulation is a constant that is independent of time and of the site of lactate measurement.
3. The LT does not pick an energetic phenomenon.
4. The higher [*La*] than at rest at powers lower than that at the MLSS is caused by early lactate accumulation during the exercise transient.

## List of symbols

CP: Critical power
$\frac{{dE}_{La}}{dt}$: Metabolic power generated by anaerobic lactic metabolism
$\frac{dLa}{dt}$: Rate of increase of lactate accumulation
${E}_{La}$: Energy derived from anaerobic lactic metabolism
[*La*]: Lactate concentration
LDH: Lactate dehydrogenase
LT: Lactate threshold
MLSS: Maximal lactate steady state
P/La ratio: Phosphate/Lactate ratio
$\dot{V}{O}_{2}$: Oxygen consumption, generic
${\dot{V}}_{m}{O}_{2}$: Muscle oxygen consumption
${\dot{V}}_{R}{O}_{2}$: Respiratory oxygen flow
$\beta$: Proportionality constant indicating the amount of energy derived from the accumulation of a unit of lactate, i.e. energy equivalent of lactate accumulation

## Footnotes

1 This means that, whenever [*La*] increases by 1 mmol per liter, an amount of energy equivalent to 2.7–3.0 ml of oxygen per kg of body mass is generated and stored as chemical energy. Since, when only glucose is used as substrate, 1 ml of oxygen provides 21.16 Joules of metabolic energy, these values correspond to 57.1–63.5 J per kg of body mass. Assuming an average body mass of 70 kg, this is equivalent to generating 3.4–3.8 kJ of chemical energy overall.
